# Sapovirus: an emerging pathogen in kidney transplant recipients?

**DOI:** 10.1007/s15010-024-02242-9

**Published:** 2024-04-09

**Authors:** Michaela Rippl, Anton Burkhard-Meier, Ulf Schönermarck, Michael Fischereder

**Affiliations:** 1 Division of Geriatrics, Department of Medicine IV, LMU University Hospital, LMU Munich, Ziemssenstr. 5, 80336 Munich, Germany; 2 Department of Medicine III, LMU University Hospital, LMU Munich, Marchioninistr. 15, 81377 Munich, Germany; 3 Division of Nephrology, Department of Medicine IV, LMU University Hospital, LMU Munich, Marchioninistr. 15, 81377 Munich, Germany

**Keywords:** Acute gastroenteritis, Sapovirus, Kidney transplant recipients, Virus infection

## Abstract

**Purpose:**

Diarrhea is an important cause of morbidity and mortality in immunocompromised patients. After including sapovirus to the viral gastroenteritis screening of our institution’s laboratory, we noticed an increase in sapovirus infections among kidney transplant recipients. Therefore, we assumed former gastrointestinal tract infections with unidentified pathogens could have been caused by sapovirus. To better understand the characteristics of a sapovirus infection in a high-risk group we initiated this study.

**Methods:**

Over a period of 6 months, all transplant recipients with diarrhea and later identified viral/unknown pathogens were included. Kidney function, levels of immunosuppressants and  c-reactive protein, acid–base balance, onset of symptoms and time of hospitalization were analyzed.

**Results:**

Among 13 hospitalized kidney transplant recipients sapovirus was detected in four patients, while in the remaining nine, three were diagnosed with norovirus, one with cytomegalovirus, one with inflammatory bowel disease and in four patients no pathogen was identified. Even though statistically not significant, creatinine levels at admission tended to be higher in sapovirus patients (median: sapovirus: 3.3 mg/dl (1.3; 5.0), non-sapovirus: 2.5 mg/dl (1.1; 4.9), *p* = 0.710). Also, Tacrolimus levels showed the same trend (sapovirus: 13.6 ng/ml (12.9; 13.6), non-sapovirus: 7.1 ng/ml (2.6; 22.6), *p* = 0.279). On discharge creatinine levels improved equally in both groups (sapovirus: 1.7 mg/dl (1.4; 3.2), non-sapovirus: 2 mg/dl (1.0; 3.6), *p* = 0.825).

**Conclusion:**

In high-risk patients, early symptomatic treatment remains crucial to protect the transplant`s function. In our cohort all patients recovered well. Larger cohorts and longer follow-up times are needed to detect the long-term consequences and a potential need for further research regarding specific treatment.

**Trial registration:**

The study has been registered on DRKS (trialsearch.who.int), Reg. Nr. DRKS00033311 (December 28th 2023).

## Introduction

Diarrhea is generally known as one of the most common diseases especially among children but also in adults. In low-income countries it is a leading cause of death due to poor general health and lack of adequate medical treatment [1]. However, also in Europe and Northern America, acute gastroenteritis defined as three or more looser unformed stools per day often presenting as watery diarrhea in combination with vomiting, is a common reason for emergency department presentation [2]. Usually, hospitalization is not necessary for immunocompetent healthy adults, whereas in immunocompromised hosts such as kidney transplant recipients diarrhea of any cause usually leads to an in-patient hospital stay requiring intravenous anti-infective therapy and search for pathogen or non-infectious causes [3, 4]. In Germany, on average 350.879 patients are hospitalized due to acute gastroenteritis every year [4, 5], and on average 2792 death per year are caused by acute gastroenteritis [4]. Among transplant recipients acute gastroenteritis is also one of the most common infections when presenting at an emergency department [5]. In a case series study about reasons for admission to emergency department in renal transplant recipients Uysal et al. identified acute gastroenteritis with a share of 11% as the number one leading cause, followed by upper respiratory tract (9%) and urinary tract infections (4%) [5].

Especially in immunocompromised hosts there are two main reasons for gastrointestinal-related symptoms, i.e., infectious and not-infectious causes.

Common pathogens of diarrhea in general are either bacterial (e.g., shigella, salmonella, E. coli, campylobacter, clostridium), viral (e.g., norovirus, enteric adenovirus, sapovirus, rotavirus, astrovirus, SARS-CoV-2) or, especially in travelers, parasites (e.g., cryptosporidia, giardia) [3]. Also in transplant recipients norovirus, adenovirus, rotavirus, and cytomegalovirus (CMV) are the leading viral infectious causes for diarrhea [6]. As sapovirus and norovirus belong to the same family (Caliciviridae) older, less specific test methods were not able to differentiate between these viruses [7]. So only with the development of more sensitive techniques, detection of previously not identified viruses—such as the sapovirus—became possible [8] and is increasingly recognized [6]. This is a crucial advancement as sapovirus was already discovered in 1976 and is since then known to be an important pathogen of acute gastroenteritis causing 2.2–12.7% of all acute gastroenteritis infections in the general population worldwide [9]. Sapovirus is a highly infectious, nonenveloped virus that is transmitted by fecal–oral-route [8]. The incubation period usually ranges from one to four days but also longer intervals are reported [8]. As in many other diarrheal diseases symptoms are usually self-limiting within one week but especially in immunocompromised patients this period can last longer and symptoms can occur with stronger intensity [8].

Nonetheless, norovirus is still the assumed leading viral cause for acute gastroenteritis in transplant recipients related to 34.8% of infections [10, 11]. Given the mentioned imprecision in detection methods in the past an actually higher share of sapovirus-infections among these could be suspected. For sapovirus-infections in transplant-recipients no valid data are yet available [6].

In transplant recipients also immunosuppressive therapy can cause diarrhea. Unfortunately, standard medication such as Mycophenolate-Mofetil (MMF) or Calcineurin Inhibitors (CNI) frequently lead to gastrointestinal complications by damaging the intestinal mucosa, inhibiting the proliferation of gastrointestinal epithelial cells or causing a release of cytokines leading to chronic inflammation [12, 13]. Especially MMF and Tacrolimus are associated with gastrointestinal complications, whereas for example Azathioprine seems to have a rather protective effect [13]. Nevertheless, also other medications (e.g., antibiotics) but also chronic diseases (e.g., cystic fibrosis or inflammatory bowel disease) and dietary- or psychosocial reasons can be the underlying causes for diarrhea [14].

Regardless of the underlying cause of gastroenteritis patients often present with severe symptoms like diarrhea, stomach cramps, fever or weight loss [3, 9]. Kidney transplant recipients are at a high risk to present with further complications such as dehydration [15] acute kidney failure [11] and Tacrolimus lapse [16].

After exclusion of bacterial causes microbiological diagnostic of viral pathogens is usually performed by stool sampling with subsequent multiplex PCR including the most common regional pathogens [17]. Regular monitoring of clinical parameters including vital signs as well as kidney function, infectious parameters and immunosuppressant serum levels is essential [18, 19].

Management of viral infections such as norovirus or sapovirus consists of rehydration, electrolyte replacement and monitoring of the patient [9]. In kidney transplant recipients, levels of immunosuppressants should be measured and a dose reduction might be considered [20]. To date, there is no approved specific treatment for viral acute gastroenteritis pathogens such as norovirus and sapovirus whereas in cytomegalovirus (CMV) infections a specific medication (Ganciclovir) already exists [19].

After sapovirus detection has been included in our institution’s routine gastroenteritis screening in the end of 2022, we noticed the detection of sapovirus in patients hospitalized due to acute diarrhea. Therefore, we presumed that earlier gastroenteritis infections which had been declared unclear might had been caused by sapoviruses as well. To evaluate characteristics of sapovirus-infections compared to other non-bacterial gastroenteritis pathogens in a high-risk group such as renal transplant recipients this study was initiated.

## Methods

### Study design and participants

Between 01/01/2023 and 30/06/2023 all kidney transplant recipients hospitalized due to diarrheal disease in which viral gastroenteritis screening was performed were retrospectively analyzed. This study was performed in line with the principles of the Declaration of Helsinki. Approval was granted by the Ethics Committee of Medical Faculty of LMU University (December 19th 2023, Number: 23-0926).

### Techniques

Stool samples were tested for norovirus and sapovirus using Multiplex Real-time PCR (Multiplex Real-time PCR by Seegene, BIO-RAD CFX96Dx, year 2019 and 2021, including norovirus 1 and 2, rotavirus, adenovirus, astrovirus and sapovirus, Allplex GI-Virus Assay: GI-9701X/100 rxns). Also, for CMV-detection an in-house-PCR methods was used. Laboratory parameters were measured in routine laboratory work-up (serum creatinine: kinetic color test based on Jaffé-method, c-reactive protein (CRP): particle enhanced immunological turbidity test, Tacrolimus levels: HPLC–MS/MS-method).

### Statistics

Statistic analyzation was performed using IBM SPSS^®^ Statistics Version 29. Baseline characteristics and other values were reported by median and 25th–75th quartile. For detection of group differences in non-parametric data the Mann–Whitney-U-Test was used.

## Results

### Study participants

**Fig. 1:**
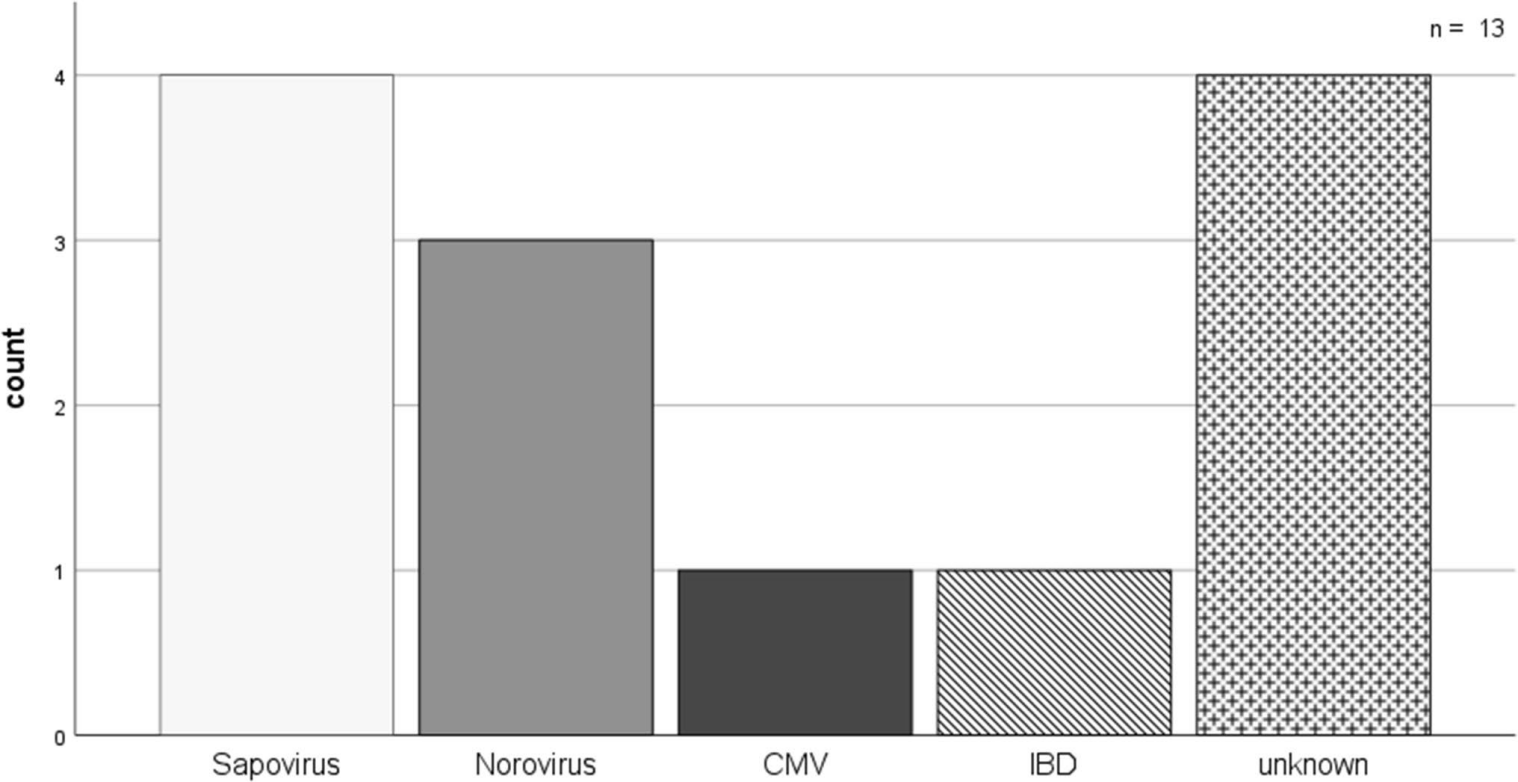
Count of identified viral or unknown pathogens among included patients (*n* = 13)

From 01/01/2023 to 30/06/2023 13 kidney transplant recipients with diarrhea later diagnosed as virally-induced or unknown pathogen were hospitalized at the division of nephrology at LMU Munich Hospital. In four patients, sapovirus was detected whereas in four other patients another viral pathogen could be detected (3 norovirus, 1 cytomegalovirus (CMV)). One patient was diagnosed with inflammatory bowel disease. In stool samples of four patients none of the tested pathogens could be detected (Fig. 1).

### Baseline characteristics

Mean age of all included patients was 51 years showing no significant differences (*p* = 0.710) between the sapovirus- and non-sapovirus groups. Onset of symptoms was reported in a median of 7 days (min. 1, max. 7) before hospitalization in the sapovirus-group, whereas first symptoms were noticed in a median of 6 days (min. 1, max. 90 d; *p* = 0.825) in the non-sapovirus group.

Mean c-reactive protein levels at admission were low in both groups (sapovirus: 0.15 mg/dl (0.1; 2.6), non-sapovirus: 0.2 mg/dl (0.1; 21.3)) and showed no significant difference between the groups (p = 0.260). Metabolic acidosis was present in 2/4 sapovirus-patients and in 3/9 non-sapovirus patients. In both groups duration of hospitalization was similar (sapovirus: 8.5 days (8; 12), non-sapovirus: 9 days (5; 20), *p* = 1.000).

Regarding renal function even though statistically not significant (*p* = 0.710) creatinine levels at admission tended to be higher (3.3 mg/dl (1.3; 5.0)) in the sapovirus-group than in the non-sapovirus group (2.5 mg/dl (1.1; 4.9)). In sapovirus patients, a median increase of 73% (− 19;144) in serum creatinine levels at hospital admission in comparison to baseline creatinine levels maximal 6 months prior to admission was noticed. In the non-sapovirus, the median increase in serum creatinine levels at admission was slightly lower with 53% ((0; 188), *p* = 0.825) (Table 2).

**Fig. 2:**
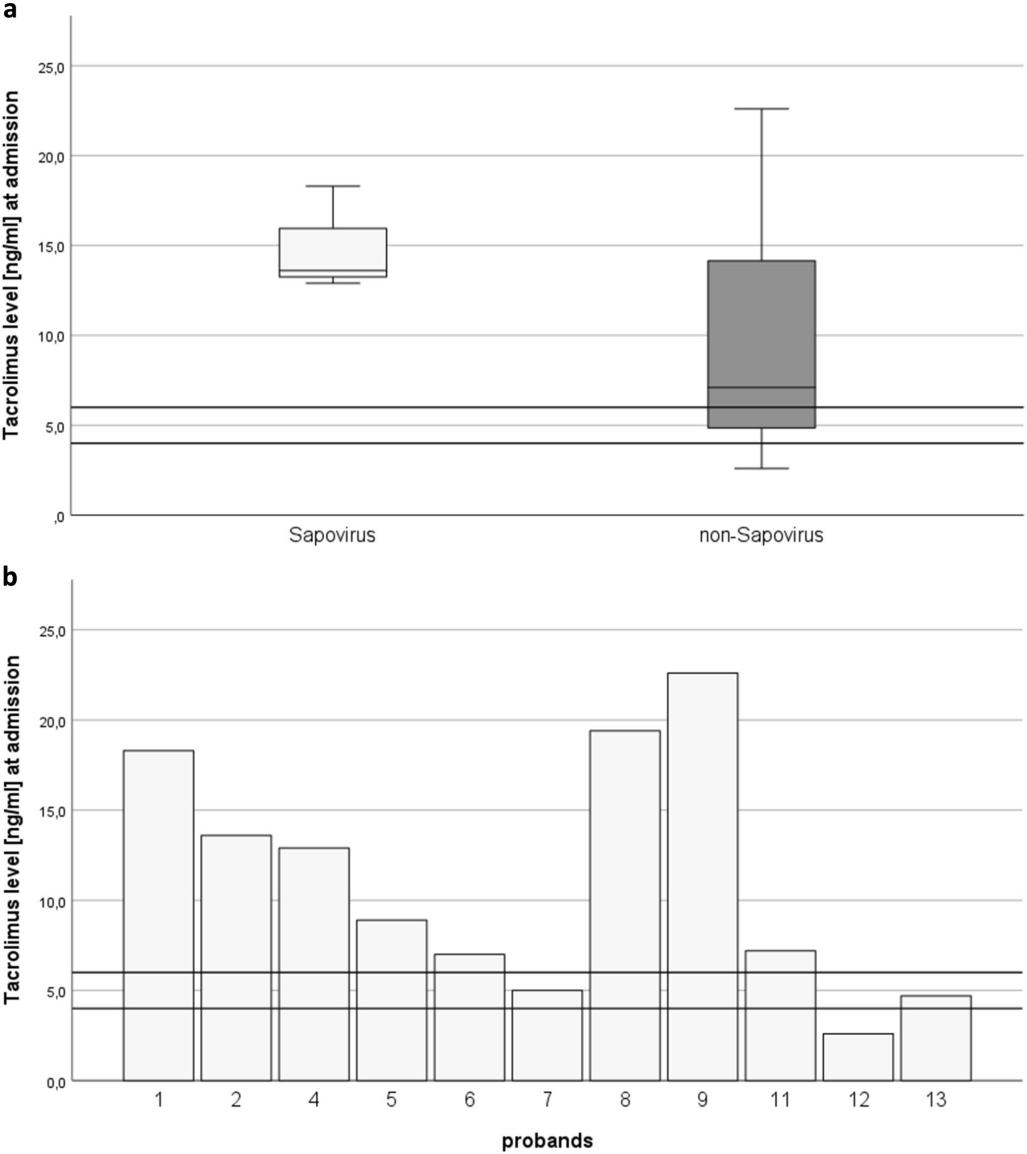
**a** Comparison of Tacrolimus-levels between patients with sapovirus infection (n = 3) vs. non-sapovirus (n = 8) at hospital admission (Tacrolimus target level  > 6 month after transplantation: 4–6 ng/ml). **b** Comparison of Tacrolimus-levels at admission by pathogens (Proband 1, 2, 4: sapovirus, proband 5, 6, 7: norovirus, proband 8: cytomegalovirus, proband 9: inflammatory bowel disease, proband 11, 12, 13: unknown pathogen; target level > 6 month after transplantation 4–6 ng/ml; proband 3 (Cyclosporin A) and 10 (Sirolimus) received different immunosuppressants)

Also, the Tacrolimus levels at admission appeared to be higher in the sapovirus-group (13.6 ng/ml (12.9; 13.6)) than in the non-sapovirus group (7.1 ng/ml (2.6; 22.6), *p* = 0.279). Overexposure was present in all sapovirus patients receiving Tacrolimus treatment. Also in two norovirus patients as well as in the patient suffering from cytomegalovirus and inflammatory bowel disease a Tacrolimus overexposure could be detected (Fig. 2b).

Kidney function improved during hospital stay in both groups (median creatinine levels at discharge sapovirus: 1.7 mg/dl (1.4; 3.2), non-sapovirus: 2 mg/dl (1.0; 3.6), *p* = 0.825).

### Overall symptoms

All patients regardless of the final diagnosis presented with ongoing diarrhea. Two patients of the sapovirus-group and three of the non-sapovirus group also reported nausea and vomiting. Also, gastric cramps and reduced overall health were repeatedly reported symptoms, especially in the non-sapovirus group. Individual patients also reported episodes of fever. Some patients were additionally suffering from oliguria leading to concerns regarding a potential rejection of the transplant.

### Treatment

No specific antiviral treatment for patients with sapovirus or norovirus infections was used. All sapovirus patients received intravenous fluids and sodium hydrogen carbonate to treat metabolic acidosis when necessary. Also, symptomatic treatment with loperamide and dimenhydrinate were applied if necessary.

In the non-sapovirus group treatment was started depending on the underlying pathogen. Because of relatively high CRP levels four patients received antibiotic treatment, assuming a bacterial infection, which was stopped after the viral pathogen diagnosis.

In the patient with cytomegalovirus-colitis treatment with Valganciclovir was initiated. In one patient diagnosed with inflammatory bowel disease Glucocorticoids, Budesonide and Azathioprine were applied. Also, one patient received Fluconazole because of a history of recent fungal infection.

### Complications

An acute decline in kidney function was observed in most patients regardless of the underlying pathogen causing diarrhea (Table 2). Diarrhea and reduction of renal function also led to acute metabolic acidosis in 5 patients (2 sapovirus, 1 norovirus, 1 inflammatory bowel disease, 1 none).

## Discussion

Diarrhea is a common disease among kidney transplant recipients. As in other populations infections with bacteria, viruses or parasites can be the underlying cause. Especially since more sensitive techniques for viral differential diagnosis developed over the last years an increase in the detection of sapovirus became possible in routine diagnostics [8]. With the implementation of sapovirus detection in our laboratory we noticed an increase of sapovirus infections.

Like norovirus the sapovirus belongs to the Caliciviridae family and due to an inaccuracy in taxonomy earlier detection methods did not differentiate between norovirus or sapovirus [8]. Therefore, we speculate that a substantial part of previous diagnosis of norovirus gastrointestinal infections might be actually caused by sapovirus. As the detection of sapovirus-infections will further increase over the next years, a profound understanding of its epidemiology, clinical significance and treatment algorithms are of high clinical relevance. To understand and highlight the characteristics of a sapovirus infection in a high-risk cohort compared to other causes of diarrhea, all kidney transplant recipients admitted to the hospital on the nephrology ward over a 6-month period between January and June 2023 and a positive viral or unidentified gastrointestinal pathogen diagnosis were included in our analysis (Fig. 1).

In all patients, except for two, acute kidney injury was detected (Table 2). The main cause was most likely prerenal caused by fluid losses due to the diarrhea leading to a reduced perfusion of the transplanted-kidney. Creatinine levels at admission and creatinine-level increase in comparison to baseline creatinine levels tended to be higher in sapovirus than in non-sapovirus patients (Table 1, creatinine level at admission sapovirus: 3.3 mg/dl (1.3; 5.0), non-sapovirus: 2.5 mg/dl (1.1; 4.9), *p* = 0.710, Table 2, sapovirus: 73% (− 19; 144), non-sapovirus 53% (0; 188), *p* = 0.825) maybe indicating higher fluid losses in sapovirus-patients than in the non-sapovirus group.

**Table 1:** Baseline characteristics

Parameters median (min; max))	Sapovirus (*n* = 4)	Non-sapovirus (*n* = 9)	*p*-value
Age [years]	52 (37; 84)	48 (19; 78)	0.710
Gender female [%]	50	44	
Number of kidney transplants	1 (1; 2)	1 (1; 3)	0.825
Onset of symptoms prior to admission [days]	7 (1; 7)	6 (1; 90)	0.727
Duration of hospital stay [days]	8.5 (8; 12)	9 (5; 20)	1.000
C-reactive protein at admission [mg/dl]	0.15 (0.1; 2.6)	0.2 (0.1; 21.3)	0.260
Tacrolimus level at admission [ng/ml]	13.6 (12.9; 13.6)^+^	7.1 (2.6; 22.6)*	0.279
Presence of metabolic acidosis at admission [%]	50	33	
Creatinine level at admission [mg/dl]	3.3 (1.3; 5.0)	2.5 (1.1; 4.9)	0.710
Creatinine level at discharge [mg/dl]	1.7 (1.4; 3.2)	2 (1.0; 3.6)	0.825

*p*-values ≤ 0.05 were considered statistically significant, ^+^: *n* = 3, *: *n* = 8.

**Table 2:** Comparison of creatinine levels at admission to baseline creatinine levels (max. 6 month prior to hospital admission) between sapovirus and non-sapovirus group

Parameters (median (min; max))	Sapovirus (*n* = 4)	Non-sapovirus (*n* = 9)	*p*-value
Absolute difference of creatinine levels at admission to baseline creatinine levels [mg/dl]	1.6 (-0.3; 2.4)	0.6 (0; 3.2)	0.604
Percentual difference of creatinine levels at admission to baseline creatinine levels [%]	73 (-19; 144)	53 (0; 188)	0.825

*p*-values ≤ 0.05 were considered statistically significant.

Also immunosuppressant`s toxicity has to be considered as a cause for kidney function decline in kidney transplant recipients suffering from diarrhea [12]. All patients infected by sapovirus who received Tacrolimus treatment and all patients except for two from the non-sapovirus group who received Tacrolimus treatment presented with a Tacrolimus overexposure (Fig. 2b). Even though not statistically significant, Tacrolimus levels were higher in sapovirus-infected patients (13.6 mg/dl (12.9; 13.6)) in comparison to the non-sapovirus group (7.1 (2.6; 22.6), *p* = 0.279) (Table 1, Fig. 2a). Due to a shorter retention time during diarrheal diseases Tacrolimus uptake is shifted to the lower gastrointestinal tract where uptake mechanisms are weaker [21], causing higher Tacrolimus blood levels during diarrheal diseases. Given the elevated Tacrolimus levels of our sapovirus-patients one could, therefore, hypothesize that the retention time might be even shorter in sapovirus-infections than in other viral pathogens explaining the higher Tacrolimus blood levels in this cohort. A larger study cohort would be necessary to test this hypothesis. Nevertheless immunosuppressants` levels should be monitored closely in transplant recipients in case of acute gastroenteritis or diarrhea regardless of the underlying reason.

For initial differentiation between bacterial and other pathogens levels of infectious parameters such as c-reactive protein are commonly used as an indicator for bacterial infections when strongly elevated [22]. In our cohort, two patients presented with strongly elevated c-reactive protein levels (21.3 mg/dl and 17.7 mg/dl) at admission but later were diagnosed with a viral (norovirus) and a non-identified pathogen. Procalcitonin and Interleukine-6 might be useful to better predict different classes of pathogens [22], unfortunately these parameters were not available in our cohort so this hypothesis could not be tested. In four patients, no underlying pathogen could be detected. These patients might have been infected by another virus that as the sapovirus earlier is not part of the routine gastroenteritis screening. Overall, also in immunocompromised patients, an advanced approach to identify the underlying pathogen (e.g., via gastro-/colonoscopy including biopsies) should be considered if symptoms cannot be cured by symptomatic treatment and when a causal treatment option after the pathogen`s identification is expected [19, 23, 24]. Therefore, after elongated symptomatic treatment that did not lead to an improvement of symptoms we decided to perform colonoscopy in one patient which lead to the diagnosis of inflammatory bowel disease.

Regarding the treatment of diarrheal diseases symptomatic treatment usually is the first option leading to a recovery of symptoms in approximately half of the patients [12]. This includes intravenous fluid substitution, hold of a diuretic therapy as well as anti-motility, anti-emetic drugs or intravenous buffer solutions if necessary.

Secondly, if available, after identification of the underlying pathogen specific treatment options should be implemented as soon as possible. However, for most viral infections including sapovirus no specific treatment is yet available. Even in norovirus infections which are very common no treatment options are available outside clinical trials. Given our cohort`s excellent recovery from sapovirus infections, without major complications that would have made permanent or intermittent hemodialysis necessary, the need for specific treatment options can be questioned. To identify the necessity for specific treatment options in sapovirus infections studies with larger patients’ cohorts and longer follow-up times are necessary. If a real need becomes apparent, given the affiliation to the same viral family (Caliciviridae) of sapovirus and norovirus, medical studies on Nitazoxanide [25–28], Immunoglobulins [29, 30] or other anti-viral drugs (Polymerase Inhibitors, Protease Inhibitors, Immunomodulators) [31] which have shown positive effects on norovirus infections should be tested.

Similar to other gastrointestinal tract infections in immunocompromised hosts, the development of chronicity is of concern [32]. Alike in norovirus-infections, also for sapovirus there are reports on chronicity and prolonged symptoms of sapovirus infections especially in immunocompromised patients [11, 33].

Recently, the occurrence of transplant rejection has been reported in two of four intestinal transplant recipients in relation to a sapovirus infection [34]. In our small study cohort of kidney transplant recipients no rejection was observed. While sapovirus infection with tropism to the small intestine might trigger a rejection directly in the intestinal transplant [34], this seems to be unlikely in kidney transplant patients, especially in the context of higher Tacrolimus exposure.

In our cohort of sapovirus patients’ age span (37–84 years) and time after transplantation (10 month–25 years) ranged widely but both time of hospitalization and improvement of kidney function were equally long and well within this group. Age and time after transplantation, therefore, might not be relevant risk factors in sapovirus infections for prolonged hospitalization or bad recovery but larger study cohorts and longer follow-up time are needed to verify this hypothesis.

A clear limitation of our study is the small sample size including a heterogeneous group of kidney transplant patients admitted to our ward. As all patients included in our study were at least 10 months after kidney transplantation, conclusions cannot be drawn regarding complications and recovery of kidney function among patients receiving higher doses of immunosuppressive medications or other organ transplantations.

Generally, due to a lack of specific treatment options and the potential of rare but severe complications general prevention should be improved [35]. For sapovirus, there is no vaccination yet available [8]. So given the fecal–oral transmission path basic hygiene measures, especially hand hygiene, are essential [35].

## Conclusion

In our small cohort of kidney transplant patients admitted to the hospital because of acute diarrhea sapovirus infection was an important differential diagnosis. Complications included a decline in kidney function and elevated tacrolimus levels. All patients improved with symptomatic treatment and kidney function returned to baseline values.

In transplant patients with acute diarrhea search for an underlying pathogen (including bacterial and viral pathogens) should be initiated as early as possible to offer optimal treatment and to reduce disease transmission. We suggest, that sapovirus should be included in the differential diagnosis.

Levels of immunosuppressives must be measured in all patients with diarrhea. Symptomatic treatment and adjustment of immunosuppressive drug dose are essential parts of the treatment. To date, there is no available specific treatment for sapovirus infections and further studies are needed.

## Data Availability

The clinical dataset will be made available upon reasonable request to the corresponding authors.
